# 

**Published:** 1917-03-03

**Authors:** 


### CONTENTS.

PAGE

**The Nation's Food and the War** ... ... ... 433

**Children's Eyesight and the Cinematograph**... 434

**Hospital and Institutional News** ... ... ... 435

The Prince of Wales' Hospital for Limbless Sailors and Soldiers—The Great Central Hotel as a Hospital for Officers—Children and Children's Hospitals—The Swansea Hospital War Loan Fund—The Way to Save—A Week of Generous Gifts—The Tom Wright Nurses' Home at Wigan — A Well-Befriended Hospital — A "Samuel Butler" Cot—Lord Knutsford's Humour?—Privileged Visiting to Union Patients —The Present Routine of Treatment at Rochester Row—A New Idea from Walton-on-Thames—A Custom Which Has Disadvantages—The Secret of Legacy-Getting—Gifts in Kind to Military Hospitals—The Shortage of Qualified House Officers—An Asylum Farm—Hospital Garden Amenities—A Tribute to a Dental Hospital—Self-Help in Wales—A Hospital that has Lost Two Presidents by Death in three Years —An Unedifying Quarrel at Cardiff—An Extravagant Scale of Diet—No Dearth of Hospital Motor Drivers—A Munition Worker and the Workhouse—The V.D. Act in Victoria—This Week's Drug Market.

**The Criminal Law Amendment Bill** ... ... 441

State Recognition of Preventive Medicine.

**Military Medicine at Waterloo** ... ... ... 442

PAGE

**The Institutional Library** ... ... ... ... 44

Prevention and Cure in Wartime—The Medical Inspection of Schools—Surgical Operations—Surgical and Gynæcological Nursing—Diseases of the Throat, Nose, and Ear, for Practitioners and Students—Physics and Chemistry for Nurses.

**The Editor's Letter-Box** ... 446

The Prevention of Venereal Disease—Hospitals and the Spirit Tax.

**Parliament and the Profession** ... ... ... 446

**The Matrons' and Sisters' Department** ... 447

The College's Barometer of Progress: The Anti-College Party Steadily Loses Ground.

Round the Hospitals.

The King Honours Homeland Workers—A Principal Matron's Advice—Nurses against Humbug and Pretence—The Central Committee's Leader Slips—A Reformed National Union?

**Nurse Training in Ireland** ... ... ... 449

Unorganised: Its Difficulties and a Pernicious System.

**Royal Red Cross** ... ... ... ... ... 450

Awards for Valuable Services in Connection with the War.

**Matrons' and Sisters' Appointments** ... ... vi

**The Institutional Worker** ... (Suppl.) 1

The Service of Patients' Dinners. Questions for March.

